# Parameterization of temperature sensitivity of spring phenology and its application in explaining diverse phenological responses to temperature change

**DOI:** 10.1038/srep08833

**Published:** 2015-03-06

**Authors:** Huanjiong Wang, Quansheng Ge, This Rutishauser, Yuxiao Dai, Junhu Dai

**Affiliations:** 1 Key Laboratory of Land Surface Pattern and Simulation, Institute of Geographical Sciences and Natural Resources Research, Chinese Academy of Sciences. Beijing, China; 2 University of Chinese Academy of Sciences, Beijing, China; 3 Oeschger Centre for Climate Change Research (OCCR) and Institute of Geography, University of Bern, Bern, Switzerland; 4 Department of Physics, New York University, New York, NY 10012, USA

## Abstract

Existing evidence of plant phenological change to temperature increase demonstrates that the phenological responsiveness is greater at warmer locations and in early-season plant species. Explanations of these findings are scarce and not settled. Some studies suggest considering phenology as one functional trait within a plant's life history strategy. In this study, we adapt an existing phenological model to derive a generalized sensitivity in space (SpaceSens) model for calculating temperature sensitivity of spring plant phenophases across species and locations. The SpaceSens model have three parameters, including the temperature at the onset date of phenophases (*T_p_*), base temperature threshold (*T_b_*) and the length of period (*L*) used to calculate the mean temperature when performing regression analysis between phenology and temperature. A case study on first leaf date of 20 plant species from eastern China shows that the change of *T_p_* and *T_b_* among different species accounts for interspecific difference in temperature sensitivity. Moreover, lower *T_p_* at lower latitude is the main reason why spring phenological responsiveness is greater there. These results suggest that spring phenophases of more responsive, early-season plants (especially in low latitude) will probably continue to diverge from the other late-season plants with temperatures warming in the future.

Plant phenology has proven to be a very sensitive indicator for climate change impacts^1^,^2^. The observed change of plant phenology across the globe became notable in responses to recent climate change^3^,^4^,^5^,^6^. The change in plant spring phenological events and life cycles of animals could significantly impact the structure and function of ecosystem, e.g. interspecific relation, carbon uptake and nutrient cycling^7^,^8^. Furthermore, the change in phenology can feed back on atmosphere through physical process (e.g. surface albedo and energy partitioning) or biogeochemical circulation (e.g. carbon cycle)^9^,^10^. A better understanding of phenology—climate interactions are crucial for climate change research.

Many studies in middle and high latitudes demonstrate that the temperature is the main driving force and interannual modulator of phenological change, while other factors (e.g. photoperiod) only play a secondary role as limiting factors^1^. Since the temperature sensitivity of plant phenological stages (phenophases change in days per degree Celsius) determines the magnitude of phenological shifts in response to future climate warming, more attention has been paid to it, both in observational records and warming experiment studies^11^.

Observed evidence shows that the temperature sensitivity of plant spring phenophases exhibited interspecific and spatial pattern^5^. Early-season plant species often exhibit stronger temperature sensitivity or faster rate of phenological change at a fixed site or across the same region^12^,^13^,^14^,^15^,^16^,^17^. With respect to the spatial pattern, the response of spring phenophases to temperature is stronger in warmer than in colder countries across Europe^4^. Likewise, in China and the United States, the plant phenophases at lower latitudes (warmer sites) show stronger temperature sensitivity^18^,^19^,^20^,^21^. An interesting question here is why phenological responsiveness to temperature is greater in early-season plant species or warmer regions. According to recent studies, the interspecific difference in temperature sensitivity may be attributed to the difference in life strategy of plant species^22^,^23^ or drivers (abiotic versus biotic) of selection on plant phenology^24^. The spatial difference of temperature sensitivity may be caused by the genetic difference due to the adaptation to local climate^25^,^26^. However, the detailed mechanisms about the interspecific and spatial pattern of phenological response to temperature remain unclear.

In the present study, we developed a model for estimating temperature sensitivity of spring plant phenophases through parameterizing temperature sensitivity from an existing phenological model. Second, using the derived model, we calculated the temperature sensitivity of first leaf date (FLD) for 20 woody plant species at 43 sites in China. Third, we discussed the interspecific variation and spatial patterns of temperature sensitivity across eastern China. Our overall objectives are to derive a generalized model for calculating temperature sensitivity of spring phenophases and use it to interpret the observed interspecific variation and spatial pattern in phenological response to temperature.

## Results

Using a phenological model based on degree day accumulation, the sensitivity in space (SpaceSens) model for calculating temperature sensitivity of spring plant phenophases was derived (equation (4), see Methods section). According to the SpaceSens model, the temperature sensitivity of the spring plant phenophase equals the length of period (*L*, which is used for performing linear regression between phenophases and temperature) divided by the difference between temperature at the onset date of phenophase (*T_p_*) and base temperature threshold (*T_b_*).

**Figure 1:**
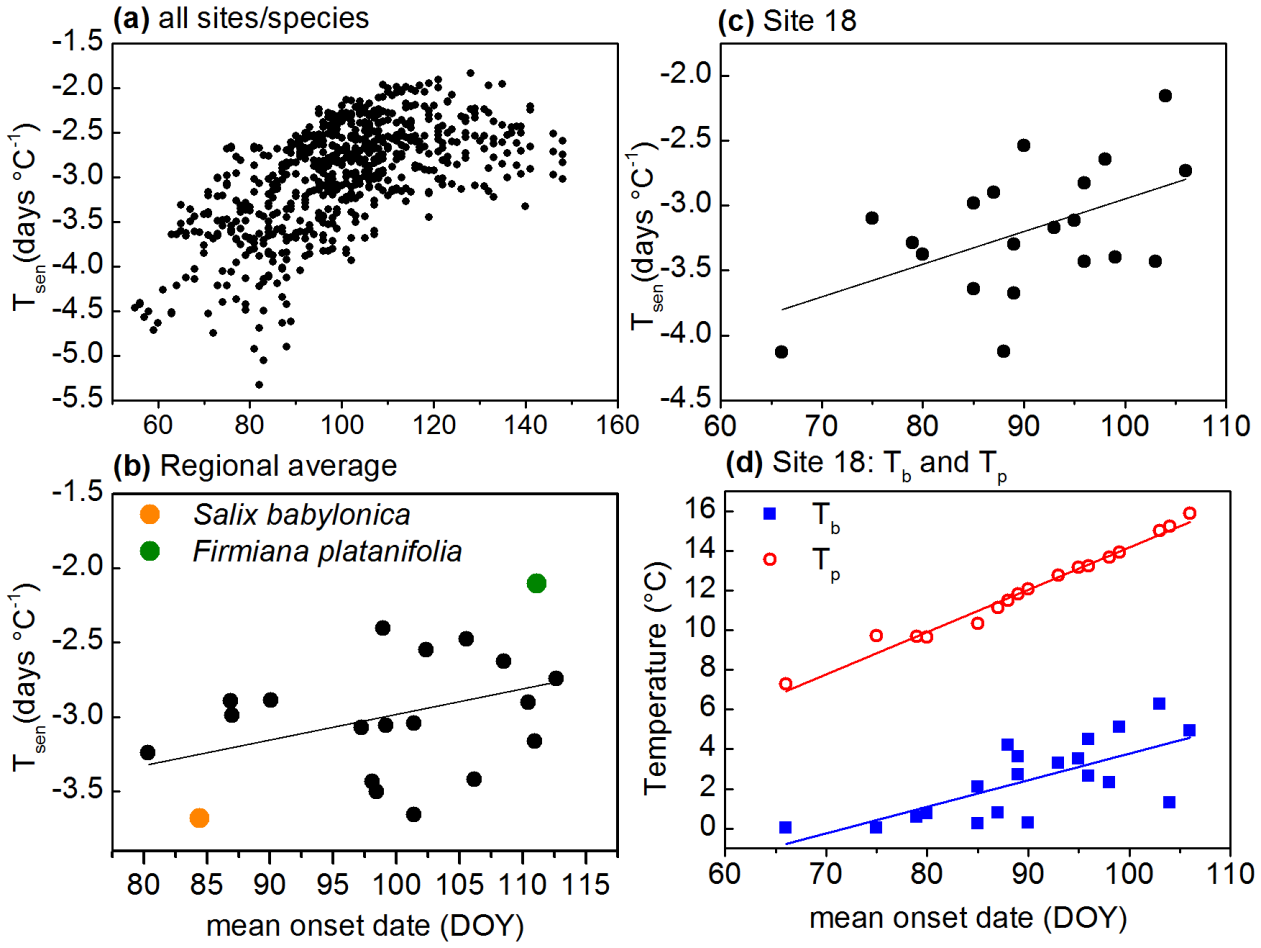
Temperature sensitivity (*T_sen_*) for first leaf date of 20 plant species plotted according to their onset date. (a) *T_sen_* for all site/species; (b) *T_sen_* for all species averaged from their distribution sites (y = 0.02x − 4.7, *R^2^* = 0.15, *P* = 0.08). Two plant species showing strongest and weakest temperature sensitivity are marked with different colors; (c) *T_sen_* for all species at site 18 (y = 0.03x − 5.46, *R^2^* = 0.26, *P* = 0.02); (d) *T_p_*: y = 0.21x − 7.16; *R^2^* = 0.98, *P* = 4.4*10^−16^; *T_b_*: y = 0.13x − 9.63, *R^2^* = 0.50, *P* = 5.0*10^−4^.

The temperature sensitivity of FLD for 20 plants species in Eastern China was simulated by using the SpaceSens model. The results show that the temperature sensitivity of FLD varies among different species and locations (Figure 1a). For example, on average, *Salix babylonica* FLD is most responsive with 3.68 days earlier for every 1°C increase in temperature of the previous month, while the response of *Firmiana platanifolia* FLD is minimum, with only 2.10 days earlier per 1°C increase (Figure 1b). Overall, the temperature sensitivity of FLD estimated from the SpaceSens model is −2.98 ± 0.61 days °C^−1^ (mean ± standard deviation, *n* = 668) for all site/species.

The early leaf unfolding time is associated with greater responsiveness to temperature variation across all sites/species, but this higher sensitivity for early-season species usually breaks down well before late-April (Figure 1a). If the FLD and its temperature sensitivity for each species is averaged over all sites, early-season species is also connected with stronger temperature sensitivity (Figure 1b, *R* = 0.38, *P* < 0.1). When considering all species at a single site, the temperature sensitivity of FLD correlated positively with their onset date in most of 43 sites (e.g. at site 18, *R* = 0.51, *P* < 0.05, Figure 1c). According to the SpaceSens model, if the *L* is constant, the temperature at the onset date of phenophase (*T_p_*) and base temperature threshold (*T_b_*) are the two factors determining the temperature sensitivity. Both *T_p_* and *T_b_* increase linearly with the onset date of FLD (taking Site 18 as an example, Figure 1d). However, the slope of *T_b_* is less than *T_p_*, which leads to the stronger temperature sensitivity for earlier phenophases.

**Figure 2:**
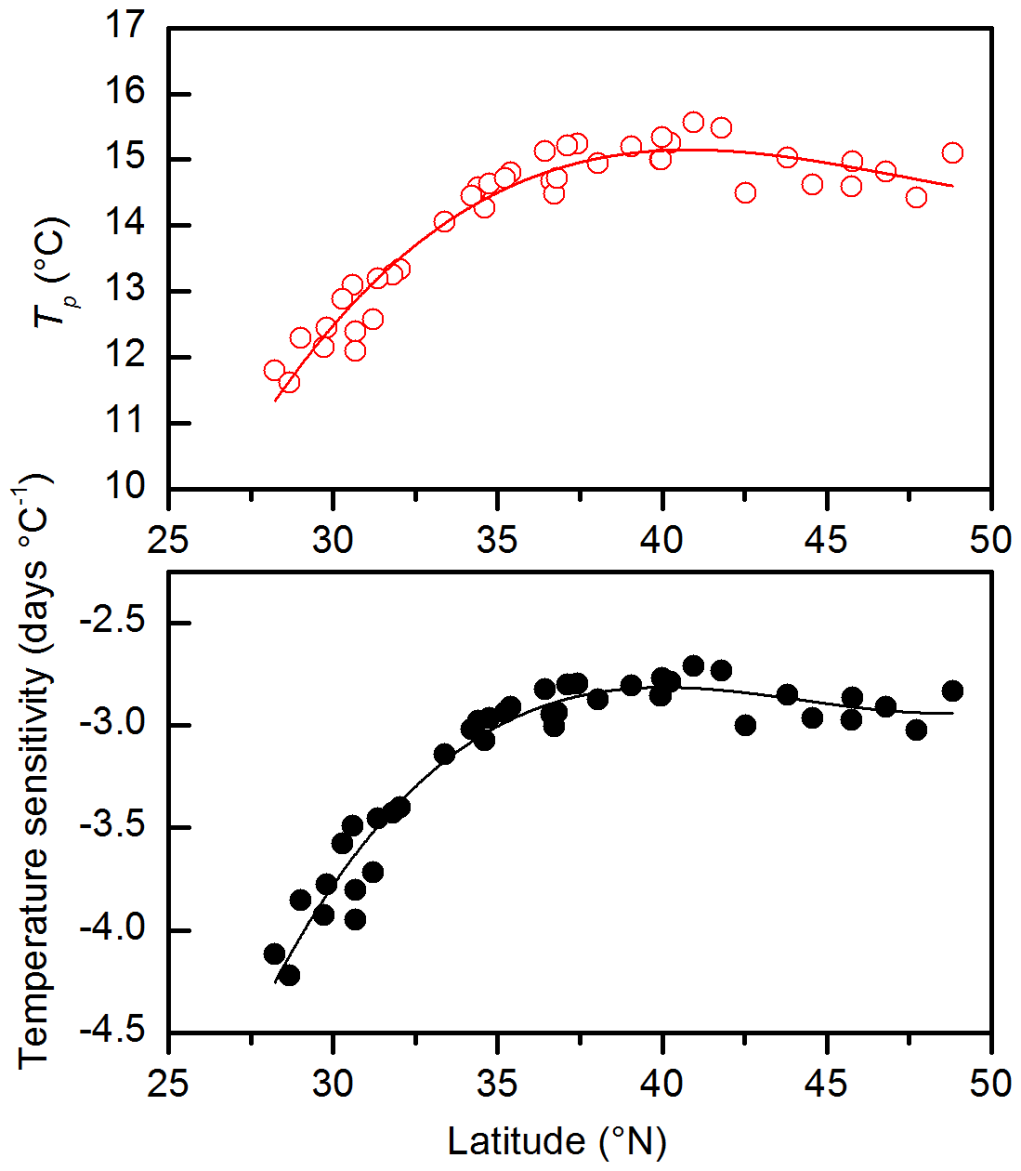
The temperature at the onset date of phenophase (*T_p_*) and temperature sensitivity (*T_sen_*) for first leaf date of *Sophora japonica* plotted according to latitude. The overall dependence of *T_p_* and *T_sen_* on latitude is high: for *T_p_*: *R^2^* = 0.93, y = −59.98 + 4.922*x − 0.1059x^2^ + 7.452*10^−4^x^3^, *n* = 41, *P* < 0.001; for *T_sen_*: *R^2^* = 0.94, y = −38.37 + 2.450*x − 0.059x^2^ + 4.204*10^−4^x^3^, *n* = 41, *P* < 0.001.

The temperature sensitivity of FLD for a specific plant species highly depends upon latitude, although the relationships are not linear (Figure S1). Generally, the temperature sensitivity of FLD is greater at lower latitude than at higher latitude. For most species, the temperature sensitivity remains stable between 40°N and 50°N (taking *Sophora japonica* as an example, Figure 2). From 40°N to 28°N, the temperature sensitivity gradually becomes stronger. According to equation (4), the spatial variation of temperature sensitivity is determined by the spatial pattern of *T_p_*, since *T_b_* and *L* remain constant among locations for a specific species. The change of *T_p_* along a latitudinal gradient is consistent with the latitudinal pattern of temperature sensitivity.

## Discussion

The magnitude of phenological response to climate change obtained by using the SpaceSens model (−2.98 ± 0.61 days °C^−1^) closely matches the results of previous studies in Europe and China^4^,^18^,^27^,^28^,^29^. All these studies showed 2.6 to 6 days advancement in spring phenological events per °C increase^5^. Therefore, the SpaceSens model gives a reliable estimation for temperature sensitivity of spring phenology.

In early spring, the plant species with earlier FLD exhibit the strongest reactions to temperature change based on the SpaceSens model. This result coincides with the results of previous studies^5^,^11^. Across late spring, sensitivities are relatively stable (Figure 1a). This pattern was also documented in a study based on a phenological dataset in Europe^30^. Some studies give several possible explanations for these findings from an ecological perspective. First, the phenological adaptation related to the life strategy of plant species could cause the interspecific difference in phenological response^22^. The short-lived, early successional species are opportunistic, and usually adopt a more risky life strategy^31^. Therefore, the phenological responsiveness of opportunistic species to temperature change is stronger than long-lived, late successional species^32^. Second, the earlier phenophases are more adapted to extreme low temperature, and thus follow more closely climate warming, while the later phenophases are more cautious to advance their spring phenology because they are more easily to be injured by late frost events^33^,^34^. Third, in mid-latitude temperate systems, biotic factors (e.g. competition between species for soil resources, light or pollinators) rather than abiotic factors (e.g. temperature) might dominate phenology during mid-season periods, when the climate is more stable than the early season^24^. This study gives an explanation from the perspective of biometeorology: *T_p_*-*T_b_* correlated positively with the onset date of FLD (because the slope of *T_b_* is less than *T_p_*, see Figure 1d), which leads to the stronger temperature sensitivity for earlier phenophases.

Since the early-season species are more responsive to temperature change, the early-flowering species may flower even earlier than the late-flowering species if the climate warming continues in the future. More dispersed flowering times across species would have important implications. For example, the pollinators, especially which rely on continuously pollen recourse, may face a challenge to survive^17^. For plant species, their competition for pollinators and patterns of hybridization may be altered^13^. However, these consequences of interspecific difference in phenological change need continued and comprehensive monitoring in the future.

The spatial patterns of temperature sensitivity in the present study supports the results that the response of spring phenology to temperature in warmer locations or at lower latitudes is stronger than in colder location or at higher latitudes^4^,^18^,^19^. The local adaptation of plant phenology to regional climate may be one reason^19^. For example, the within-species variations of phenological response to temperature in Japan were less in the plant populations with lower genetic diversity^26^. More studies comparing the phenological responses between cloned plants and natural plants will be useful for quantifying the degree of genotypic difference affecting phenological responses. Our study indicated, even though there is no genotypic difference within species, the lower *T_p_* at lower latitude (warmer locations) also can explain the spatial variation of phenological responsiveness to temperature (Figure 2). However, another study found that the observational sensitivities of phenology were not associated with latitude^11^. We noticed that the phenological observation data in reference 11 was mainly from the area between 40°N and 60°N, where the *T_p_* is stable (Figure 2). If their study area is expanded to more southern area, the temperature sensitivity of spring phenophases would increase.

The spatial pattern of temperature sensitivity is a considerable factor impacting temporal trends of plant phenophases across space. If the increase in temperature is constant across space, the phenological trends would be weaker in higher latitude, since the temperature sensitivity is weaker there. Actually, higher latitude has warmed more than the lower latitude over the past 100 years^35^. Thus the effect of weaker temperature sensitivity and stronger warming trend in high latitude could partially offset each other, which make the latitude not an important factor influencing the phenological trends^36^.

Furthermore, the observed evidence demonstrates that temperature sensitivity changes with time for specific plant phenophases^37^,^38^. For example, the response to a 1°C increase in spring (March–May) temperature during a 30-year shifting time window was −2.5 to −5 days in Switzerland and −2.5 to −15 days in the UK from 1753 to 1958^39^. In Beijing, China, the temperature sensitivity of first flowering date for 20 species during the 1990–2007 are shown to be greater than during the previous period (1963–1989), with a difference of −0.87 days per 1°C on average^15^. In this study, we use the average climate from 1961 to 1990 as the normal scenario to calculate temperature sensitivity. If using climate in other periods, the estimated temperature sensitivity is expected to be different. So the temporal shift of temperature sensitivity also can be explained by employing our SpaceSens model.

A prevalent decline in the temperature sensitivity of spring phenology with increasing local spring temperature variance was found at the species level from ground observations^40^. Through our analysis, local spring temperature variance (the multiyear averaged standard deviation of daily detrended air temperature from March to May) correlated significantly and positively with latitude (*R* = 0.49, *P* < 0.01, Figure S2). Therefore, higher latitude was associated with greater spring temperature variance and lower temperature sensitivity. This explains the result that temperature sensitivity declines with increasing local spring temperature variance^40^.

## Methods

### Model development

We adapt an existing phenological model to derive a generalized description of temperature sensitivity of spring plant phenophases. The degree day model (also called thermal time model) based on the linearity between development rate of plant and temperature has been well used in phenological studies^41^,^42^,^43^. The equation of the degree day model is:
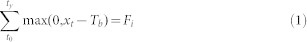
where *x_t_* is the daily mean temperature at the day *t*. *T_b_* is the base temperature threshold for temperature accumulation. *F_i_* is the critical value of degree days for onset of the plant phenophase at site *i*. *t_y_* is the onset date of the plant phenophase. *t_o_* is the start date of the degree days calculation. The degree day model implicitly assumes that environmental conditions (e.g. chilling requirement or day length) required to break dormancy have been met before *t_0_*^41^.

The degree day model of a specific plant can only be applied at a single site, since the critical value of degree days sum for onset of the plant phenophase is different among different locations^44^. The previous experiments showed that degree day required to budburst decreases exponentially with increased amount of previous chilling^43^,^45^,^46^,^47^. Therefore, *F_i_* can be described as an exponential function of coldness of the winter^48^:
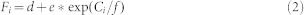
where *d, f, e* are constant parameters. *C_i_* is the mean winter temperature (December to February) at site *i* during a reference period (1961–1990). Equation (1) and (2) constitute the spatio-temporal phenological model.

**Figure 3:**
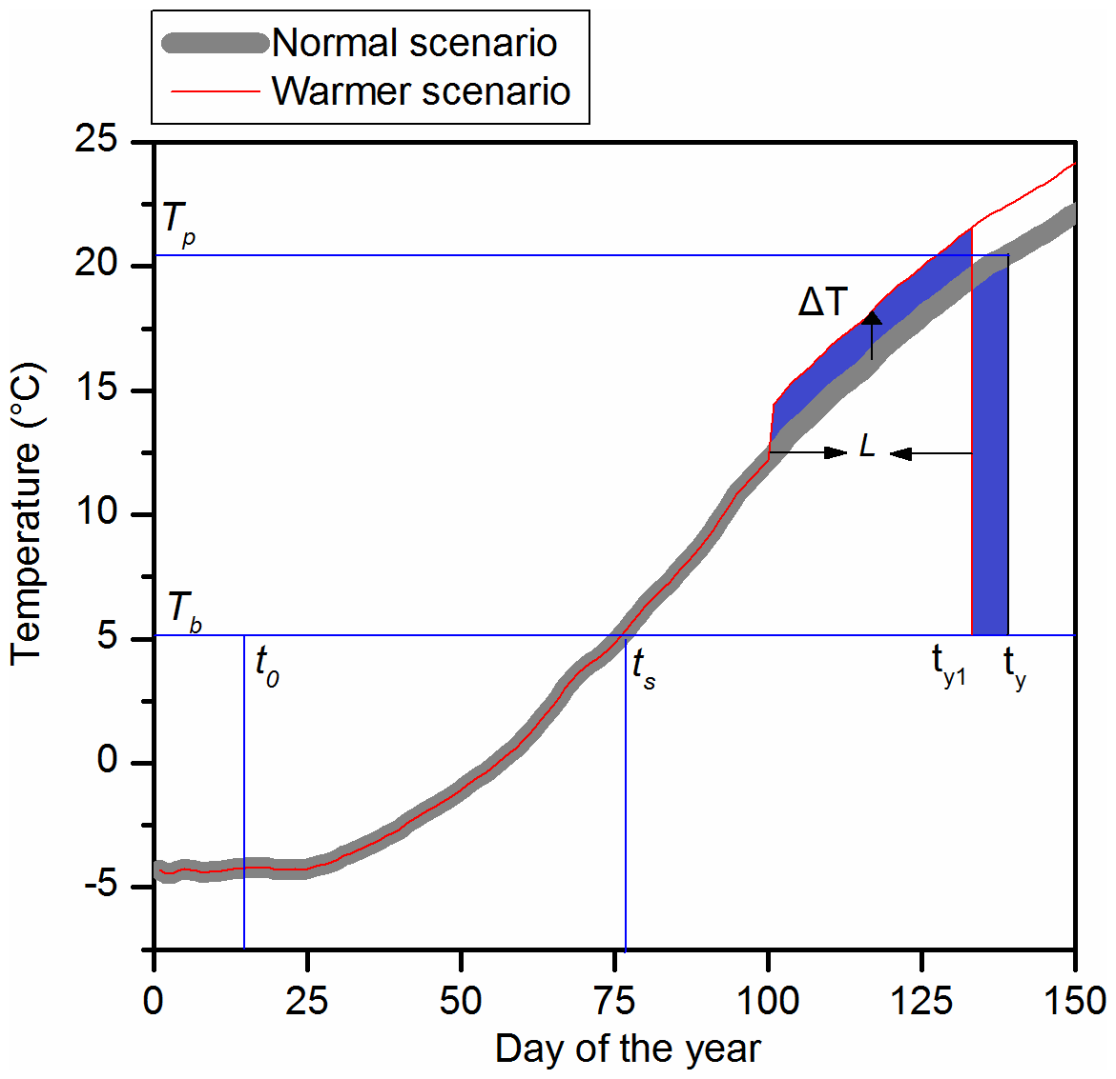
Scheme of sensitivity in space (SpaceSens) model. Bold gray line represent normal temperature scenario in reference period and thin red line represent warmer scenario (a specific period with a length of *L* before phenophases increased by ΔT, ΔT → 0). *t_0_*: the staring date of the degree day calculation; *T_b_*: base temperature threshold; *t_y_*: onset date of the plant phenophase in the normal scenario; *t_s_*: the day when temperature exceed *T_b_*; *T_p_*: temperature at the day *t_y_*; *t_y1_*: the onset date of plant phenophase in the warmer scenario.

In phenological studies, the temperature sensitivity of phenophases is usually measured as the slope coefficient of a linear regression model with phenophase as the dependent variable and temperature during a specific period before the mean phenophase as the independent variable. According to the principle of the spatio-temporal model (Figure 3), if the temperature during above-mentioned specific period (with a length of *L*) increased by ΔT (ΔT → 0), the increased degree days due to the temperature rise and the decreased degree days due to phenological advance should be equal (i.e. the areas of the two blue regions shown in Figure 3 should be equal). The equation could be expressed as follows:
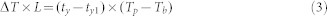
where *t_y_* is the onset date of the plant phenophase in the normal scenario. *T_P_* is the temperature on date *t_y_*. *t_y1_* is the onset date of plant phenophases in the warmer scenario. *L* is length of period, which is used for performing linear regression between phenophases and temperature (note that *L* is usually less than *t_y_* − *t_s_*, where *t_s_* is the day when the temperature exceed *T_b_*).

Temperature sensitivity of phenophases (*T_sen_*) can be regarded as the number of days changed in phenophases (Δt) divided by the change in temperature in degrees Celsius (ΔT). According to equation (3), the equation for Δt/ΔT is derived:
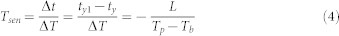
where *T_sen_* is the temperature sensitivity of the plant phenophase; the meanings of three parameters (*L*, *T_b_* and *T_p_*) are same with equation (3). Hereafter, we call equation (4) sensitivity in space (SpaceSens) model.

### Data source and statistical analysis

**Figure 4:**
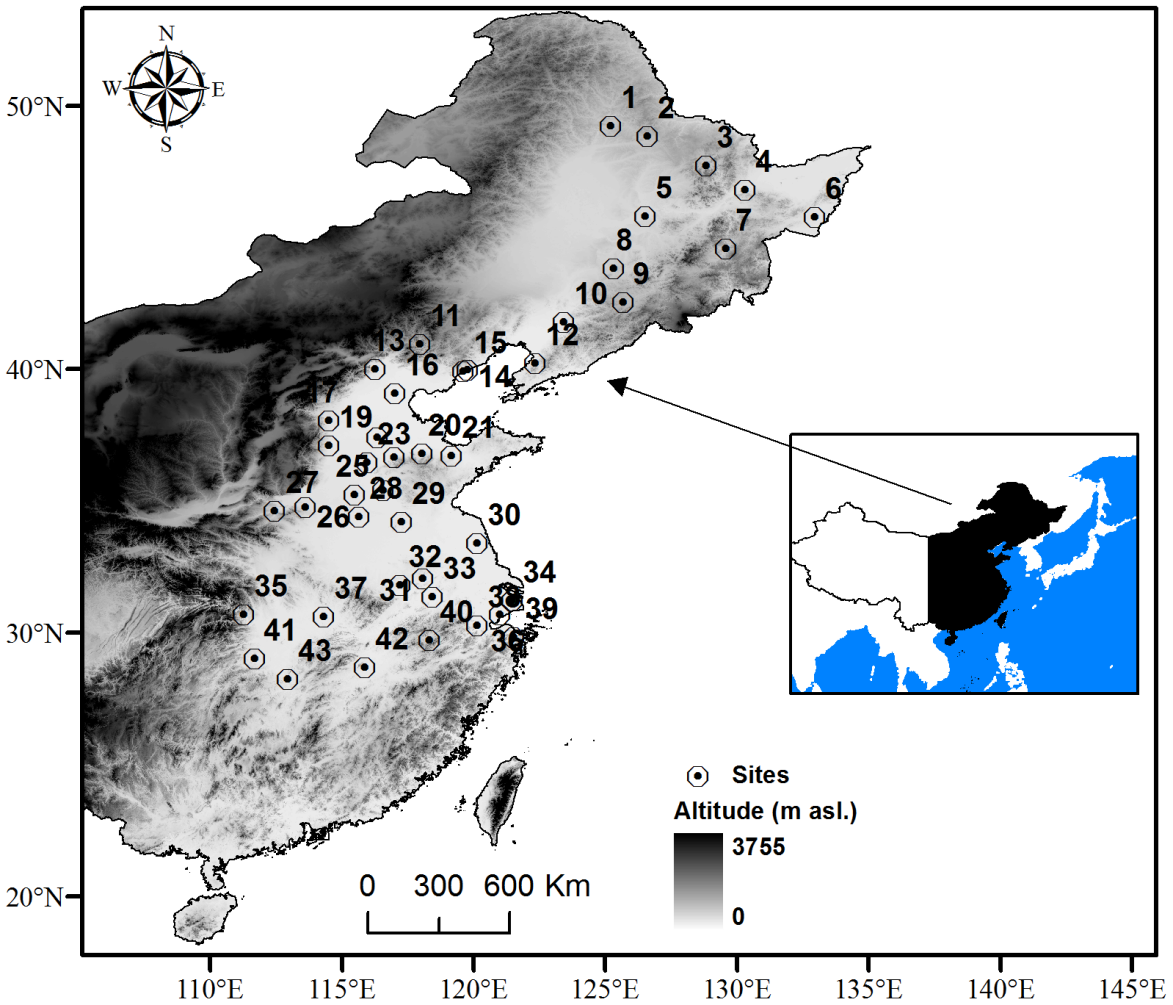
Locations of 43 phenological observation sites for studying the spatial pattern of temperature sensitivity. The software ArcGis 9.3 was used to create the map.

A total number of 5004 FLD observations for 20 woody plants from 43 sites with altitude less than 350 meters a.s.l. in eastern China were selected for calibration of the phenological models (Figure 4). The phenological data (1963–2009) was from the China Phenological Obsevation Network (CPON, http://www.cpon.ac.cn/). Phenology of each species was observed at 11 to 23 sites. The phenological time series have various lengths at different sites ranging from 1 to 37 years. According to the obsevation criteria of CPON, FLD was defined as the date when a individual formed a full first leaf. Usually, for each plant species, only one individual was observed at each site. If more than one individual is observed, the reported date is the average among all individuals of the same species. The sites span a wide latitudinal range (between 28°N and 49°N) and have a temperate or subtropical monsoon climate^49^. The daily mean temperature data from 1961 to 2009 at these sites were downloaded from the China Meteorological Data Sharing Service System (http://cdc.cma.gov.cn/cdc_en/home.dd).

Because the dataset from CPON included more than 600 plant species, we selected a number of typical woody species following these specific criteria: (1) the species must be a widespread plant which has been observed at more than 10 sites, because we want to study the spatial pattern of phenological responses; (2) the data of a species have as many observations as possible in order to calibrate the phenological models more accurately. As a result, 20 widespread plant species which have most abundant data were selected to investigate the interspecific and spatial difference of temperature sensitivity (Table 1). The current distribution range of each plant species was taken from a comprehensive atlas of woody plants in China^50^. Model parameters (*t_0_*, *T_b_*, *d*, *e*, *f*) of spatio-temporal phenological model for simulating first leaf dates (FLD) were calibrated by Ge *et al*.^48^. External validation with independent observations in Ge *et al*.^48^ indicates that the model simulates FLD with a mean root-mean-square error (RMSE) of 6 days (see Table S1). The annual FLD of these 20 woody plants at sites within their present distribution range were simulated applying the model parameters in Table S1. The mean FLD of these species across their distribution sites all occurred in spring (from 20 March to 21 April, Table 1).

For caculating temperature sensitivities, daily mean temperature time series were averaged over the reference period 1961–1990 for each site. Such daily mean temperature series can be regarded as temperature in the normal scenario (see Figure 3). The methods of estimating three variables in SpaceSens model were the following:

1. *T_p_* is a variable, which depends on species, sites and climatological mean. Firstly, we calculated the onset date of FLD (*t_y_*) in the normal scenario for each species/site by using the spatio-temporal phenological model described in Ge et al^48^ (see above). Then *T_P_* was measured as the temperature at *t_y_* (Figure 3).
2. *T_b_* is a species-specific variable, which was given through calibration of model parameters (Table S1).
3. Because our goal was to compare the pattern of temperature sensitivity derived from our SpaceSens model with that derived from regression analysis, the value of *L* should follow the convention in phenological studies based on regression analysis. For most studies, the length of specific period (*L*) considered for regressing phenology against temperature is one (or two) month or a number of days before the mean phenophases, and remained the same for all species and at all sites^4^,^13^,^14^,^51^. Thus we set *L* to 30 days as commonly used. In addition, since *L* did not vary with species and sites, the value of *L* would not affect the spatial and interspecific patterns of temperature sensitivity.

Consequently, the temperature sensitivities of FLD for each species/site could be estimated by using equation (4). At last, the relationship between the temperature sensitivity and FLD of 20 plant species at a fixed site and the relationship between the temperature sensitivity of FLD and latitude for a specific plant species were interpreted through regression analysis.

## Author Contributions

H.W. and J.D. designed the research, H.W., Q.G. and R.T. contributed to interpreting the results and writing the main text. Y.D. helped to modify the model H.W. prepared the figures. All authors reviewed the manuscript.

## Supplementary Material

Supplementary Information Supplementary Information

## Figures and Tables

**Table 1:** Twenty plant species, their distribution ranges and first leaf date (FLD) averaged from sites within respective distribution range in eastern China. N: the latitude at the northern range limit; S: the latitude at the southern range limit. SD: standard deviation of FLD across sites within respective distribution range

No.	Species	Scientific name	FLD (month/date)	SD (days)	N	S
1	Chinese ash	*Fraxinus chinensis*	4/10	17.9	46	22
2	Ailanthus	*Ailanthus altissima*	4/17	16.1	46	22
3	Chinaberry tree	*Melia azedarach*	4/15	7.6	39	19
4	Dragon tree	*Paulownia fortunei*	4/7	7.8	35	20
5	Wild apricot	*Armeniaca vulgaris*	4/6	17.8	45	22
6	Early lilac	*Syringa oblata*	3/27	14.1	42	29
7	Goldenrain tree	*Koelreuteria paniculata*	4/7	15.6	43	24
8	Rose of sharon	*Hibiscus syriacus*	4/8	12.2	40	19
9	White mulberry	*Morus alba*	4/19	19.2	49	19
10	Chinese parasol tree	*Firmiana platanifolia*	4/20	10.8	41	19
11	Siberian elm	*Ulmus pumila*	4/11	20.4	54	23
12	Chinese redbud	*Cercis chinensis*	3/26	10.8	36	23
13	Peach	*Amygdalus persica*	3/20	11.0	36	19
14	Pagoda tree	*Sophora japonica*	4/19	18.7	49	22
15	Persian silk tree	*Albizia julibrissin*	4/21	11.2	41	20
16	Paper mulberry	*Broussonetia papyifera*	4/14	11.0	41	19
17	Weeping willow	*Salix babylonica*	3/24	21.8	48	19
18	Maidenhair tree	*Ginkgo biloba*	4/7	12.3	42	25
19	Chinese wingnut	*Pterocarya stenoptera*	3/30	16.7	42	22
20	Persian walnut	*Juglans regia*	4/10	15.0	45	29
