# Supplementary material for: Parameterization of temperature sensitivity of spring phenology and its application in explaining diverse phenological responses to temperature change
Source: Sci Rep. 2015 Mar 6;5:8833. doi: 10.1038/srep08833 (PMC4351518; doi:10.1038/srep08833)
Supplement: Supplementary Information [file srep08833-s1.pdf]

Supplementary materials for:

Parameterization of temperature sensitivity of spring phenology and its application in explaining diverse phenological responses to temperature change

Huanjiong Wang [1, 2], Quansheng Ge[1], This Rutishauser[3], Yuxiao Dai[4], Junhu Dai[1]*

[1] Key Laboratory of Land Surface Pattern and Simulation, Institute of Geographical Sciences and Natural Resources Research, Chinese Academy of Sciences. Beijing, China

[2] University of Chinese Academy of Sciences, Beijing, China

[3] Oeschger Centre for Climate Change Research (OCCR) and Institute of Geography, University of Bern, Bern, Switzerland

[4] Department of Physics, New York University, New York, NY 10012, USA

Correspondence and requests for materials should be addressed to J.D. (daijh@igsnrr.ac.cn) or Q.G. (geqs@igsnrr.ac.cn)

Table S1 Parameters, internal and external validation of the spatio-temporal models for simulating FLD of 20 species.

species	Model parameters					Internal validity			External validity		
	d	*e*	*f*	T_b	t_0	N1	*RMSE*	R^2	N2	*RMSE*	R^2
Fraxinus chinensis	170.2	15.60	4.170	4.211	8	100	5.16	0.92**	90	5.52	0.89**
Ailanthus altissima	270.3	43.01	4.893	2.634	30	141	4.69	0.87**	133	4.86	0.85**
Melia azedarach	175.4	34.94	7.787	6.284	17	214	6.68	0.85**	187	6.54	0.85**
Paulownia fortunei	197.2	30.49	5.679	5.101	14	161	6.47	0.84**	167	6.18	0.83**
Armeniaca vulgaris	232.1	30.65	7.336	2.079	9	204	5.92	0.90**	194	6.04	0.90**
Syringa oblata	237.6	20.80	5.160	0.01172	23	157	6.53	0.89**	152	6.83	0.89**
Koelreuteria paniculata	170.2	48.06	4.747	3.634	2	105	5.97	0.81**	97	5.65	0.81**
Hibiscus syriacus	230.8	40.91	5.618	3.308	7	98	6.86	0.85**	92	5.97	0.86**
Morus alba	250.1	21.73	6.309	3.524	15	92	6.33	0.88**	85	6.00	0.87**
Firmiana platanifolia	338.8	149.05	9.958	1.300	30	132	6.36	0.61**	123	5.86	0.68**
Ulmus pumila	256.4	63.13	6.950	0.2580	29	182	6.54	0.83**	180	6.05	0.85**
Cercis chinensis	234.6	132.1	8.346	0.7848	6	115	6.53	0.81**	105	5.78	0.83**
Amygdalus persica	232.3	65.80	6.942	0.7653	9	99	6.89	0.87**	90	5.44	0.91**
Sophora japonica	173.0	58.13	6.126	4.505	12	105	6.46	0.65**	108	6.48	0.60**
Albizia julibrissin	215.2	96.34	6.831	4.907	1	84	6.17	0.64**	78	6.80	0.60**
Broussonetia papyifera	339.8	17.69	9.816	2.315	30	64	6.88	0.83**	62	5.44	0.87**
Salix babylonica	170.0	15.11	4.349	0.01776	22	240	6.20	0.92**	229	6.96	0.90**
Ginkgo biloba	310.8	66.20	6.01	0.2737	30	132	6.70	0.73**	118	6.79	0.75**
Pterocarya stenoptera	282.3	23.60	9.850	0.5577	7	60	5.92	0.91**	55	5.72	0.92**
Juglans regia	181.3	63.05	8.231	2.704	25	91	5.69	0.84**	83	5.11	0.86**

d, e, f, T_b, t_0: specific parameters for the spatio-temporal phenological model; N1, N2: number of observations for internal and external validity, respectively; R^2: variance explained by the model; **: $P<0.01$; *RMSE*: root mean square error. The data derived from Ge et al. (2014) ,Int J Biometeorol, 58(4), 473-484.

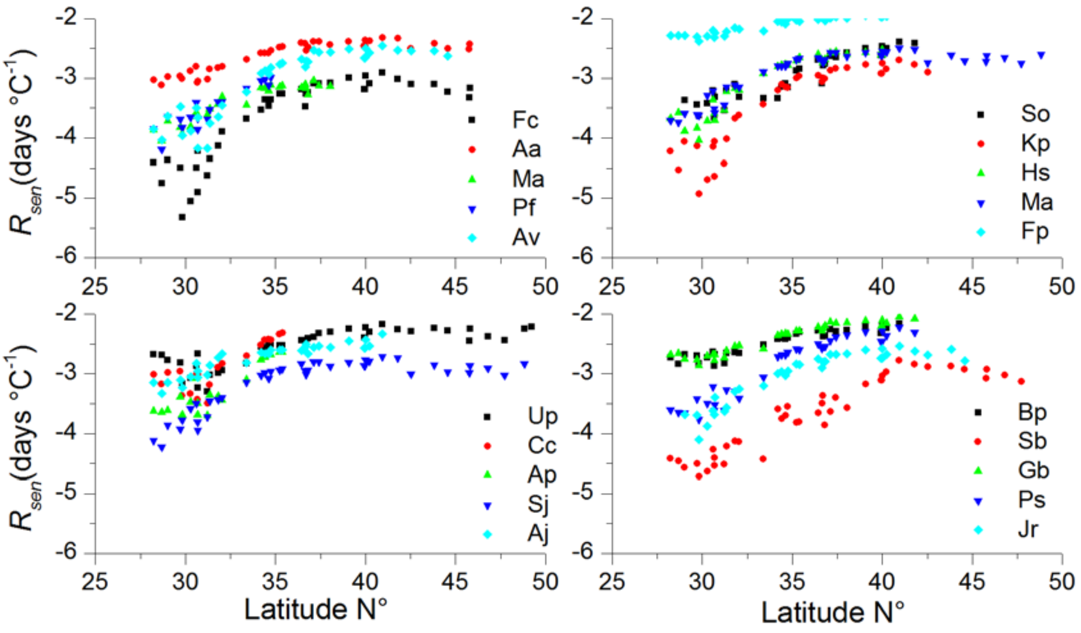

Figure S1 The temperature sensitivity (R_{sen}) for first leaf dates of 20 plant species plotted according to latitude. The scientific name of each species in the figure is abbreviated (e.g. *Fc* represents *Fraxinus chinensis* in Table 1).

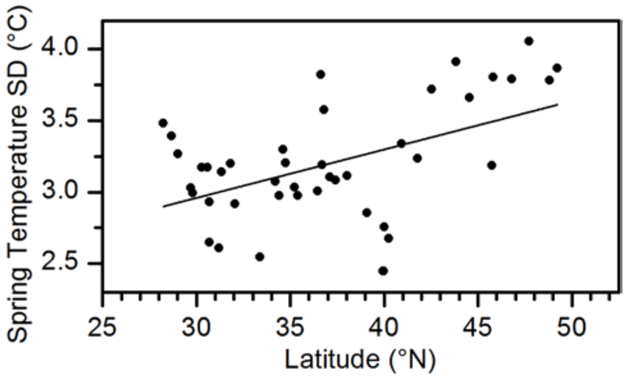

Figure S2 The relationship between the multiyear (1961-1990) averaged standard deviation (SD) of daily detrended air temperature from March to May (representing local spring temperature variance) and latitude of 43 sites (R=0.49, P<0.01)
